# Action needed to improve equity and diversity in global eye health leadership

**DOI:** 10.1038/s41433-020-0843-y

**Published:** 2020-03-20

**Authors:** Aryati Yashadhana, Justine H. Zhang, Sumrana Yasmin, Priya Morjaria, Peter Holland, Hannah Faal, Matthew J. Burton, Jacqueline Ramke

**Affiliations:** 1 School of Public Health and Community Medicine, University of New South Wales, Sydney, NSW Australia; 2 International Centre for Eye Health, London School of Hygiene & Tropical Medicine, London, UK; 3 Manchester Royal Eye Hospital, Manchester, UK; 4 Sightsavers, Islamabad, Pakistan; 5 International Agency for the Prevention of Blindness, London, UK; 6 Department of Ophthalmology, University of Calabar, Calabar, Nigeria; 7 Moorfields Eye Hospital, London, UK; 8 School of Optometry and Vision Science, University of Auckland, Auckland, New Zealand

**Keywords:** Scientific community, Business and industry

Four out of every five people with vision loss live in low- and middle-income countries (LMIC) [1], and within all countries, good vision and eye health are not experienced equally. Eye health inequity is sustained and reproduced by interlinking factors including gender [2], ethnicity [3], and socioeconomic status [4]. Women in LMICs tend to have poorer access to eye care due to socially constructed gender norms that limit their mobility and financial decision-making power [5–7]. In high-income countries (HIC), services are less accessible to ethnic-minority groups [8, 9] and Indigenous peoples [10], and subsequently these groups are disproportionately afflicted by vision impairment and blindness [1, 3, 5].

Organisations that are more diverse are more likely to consider otherwise marginalised perspectives, including by promoting gender and ethnic-minority focused agendas and knowledge creation [11]. Further, when more women are involved in board and senior management positions in the corporate sector, board development activities increase [12], conflict decreases [12], and social responsiveness is stronger [13]. Thus, global eye health organisations that place women, and people from ethnic minority and LMIC backgrounds in leadership positions are likely better placed to respond to the unequal distribution of eye health and care between and within countries [14].

In common with health and development organisations more broadly, eye health providers and eye health organisations are predominantly female, but leadership roles tend to be held by men [14, 15]. Recently, more attention has been paid to the imbalance of gender [14, 16] and ethnic representation [15] among health, development, and academic organisations.

To inform the ongoing *Lancet Global Health* Commission on Global Eye Health [17], we recently explored gender parity and ethnic diversity among leaders of the 150 member organisations comprising the International Agency for the Prevention of Blindness (IAPB), the overarching alliance for the global eye care sector. Between November 2019 and January 2020, we were able to retrieve information from the websites of 119/150 organisations. From these websites, we manually extracted all available names of the board members, chairperson, senior management staff, and chief executive officer (CEO), and where a written gender pronoun or photo was available, we assessed and manually recorded their gender and ethnic-minority status. We used validated software tools to assign gender (Gender-API, version 3.14) and ethnicity (Onolytics, 2020 version) to each person based on their name. Our manual assessment agreed with the software in 93% and 90% of cases for gender and ethnicity, respectively, and, using either method, only one person was unable to be assigned a gender and five people were not assigned ethnicity status. We acknowledge the limitation that both the software and our assessment of photographs may not have accurately determined how a person identifies; however, this limitation is unlikely to have introduced systematic bias. We calculated the proportion of position holders who were women, and in organisations based in Australasia, North America, and Western Europe, the proportion of positions held by people from an ethnic minority.

Globally, fewer than one in five chairs (17.8%) and one in three board positions (28.3%) were held by women (Table 1). Compared with board and chair positions, women were more commonly found amongst senior management teams (40.9%) and CEOs (34.5%), but still not close to parity.

**Table 1:** Proportion of boards, senior management teams, chairs and CEOs of member organisations of the International Agency for the Prevention of Blindness (IAPB) who are women, 2020.

GBD Super-regions	Organisations			Board		Senior management		Chair		CEO	
	Data available *n*	No data available^a^ *n*	Indivduals included *n*	Female *n* (%)	Total *n*	Female *n* (%)	Total *n*	Female *n* (%)	Total *n*	Female *n* (%)	Total *n*
High income^b^	85	12	1083	255 (30.7)	830	119 (45.2)	263	14 (21.2)	66	23 (37.7)	61
Latin America and Caribbean	2	11	30	3 (14.3)	21	6 (66.7)	9	0	2	0	2
North Africa and Middle East	4	2	30	6 (25.0)	24	2 (33.3)	6	0	6	1 (33.3)	3
South Asia^c^	21	2	252	34 (18.1)	188	8 (12.5)	64	1 (7.1)	14	3 (21.4)	14
South-East Asia, East Asia and Oceania	3	4	29	11 (50.0)	22	2 (28.6)	7	1 (50.0)	2	0	1
Sub-Saharan Africa	4	–	41	3 (15.8)	19	12 (54.5)	22	0	2	2 (66.7)	3
Total	119^d^	31	1465	312 (28.3)	1101	149 (40.9)	364	16 (17.8)	90	29 (34.5)	84

*CEO* chief executive officer, *GBD* global burden of disease.

^a^Some of these organisations had Facebook pages and others were academic departments with no data on board/management team members.

^b^Includes organisations from the regions: Asia Pacific, Australasia, Southern Latin America, North America and Western Europe.

^c^Includes one person of unknown gender.

^d^Information not available for all leadership categories across organisations.

IAPB organisations are predominantly situated in the HIC regions of Australasia, North America and Western Europe. Countries in these regions have considerable ethnic-minority populations—for example, 40% of people in the United States [18], 34% in New Zealand [19], 15% in Australia [20] and 14% in the UK [21] identify with at least one non-European ethnicity. Significant resource mobilisation for eye health occurs in these regions, allowing leaders of organisations to drive the global agenda, including distribution of funding and resources to LMICs. In these regions, gender parity was only observed amongst senior management teams in Australasia (Table 2). For all roles across all three regions, ethnic-minority women were the group holding fewest leadership positions, and North America was the only region in which they were a board chair or CEO (Table 2).

**Table 2:** Board members, senior management teams, chairs and CEOs of 75^a^ member organisations of the International Agency for the Prevention of Blindness (IAPB) in Australasia, North America and Western Europe disaggregated by gender and ethnic minority status, 2020.

Region	Non-ethnic minority men		Ethnic minority men		Non-ethnic minority women		Ethnic minority women	
	*n*	Row %	*n*	Row %	*n*	Row %	*n*	Row %
Australasia								
Chair	5	62.5	0	0.0	3	37.5	0	0.0
CEO	5	55.6	0	0.0	4	44.4	0	0.0
Board	47	49.5	9	9.5	32	33.7	7	7.4
Senior management	9	40.9	1	4.5	11	50.0	1	4.5
North America								
Chair	18	66.7	4	14.8	3	11.1	2	7.4
CEO	13	50.0	4	15.4	7	26.9	2	7.4
Board^b^	229	50.6	88	19.4	92	20.3	42	9.3
Senior management^c^	43	35.8	18	15.0	43	35.8	15	12.5
Western Europe								
Chair	18	81.8	1	4.5	3	13.6	0	0.0
CEO	12	54.5	2	9.1	8	36.4	0	0.0
Board^d^	144	62.3	13	5.6	61	26.4	10	4.3
Senior management	34	41.0	11	13.3	33	39.8	5	6.0
All three regions								
Chair	41	71.9	5	8.8	9	15.8	2	3.5
CEO	30	52.6	6	10.5	19	33.3	2	3.5
Board	420	53.9	110	14.1	185	23.7	59	7.6
Senior management	86	38.2	30	13.3	87	38.7	21	9.3

^a^Organisations in high income regions of Southern Latin America and Asia Pacific not included (*n* = 10).

^b^Two individuals with unknown ethnicity.

^c^One individual with unknown ethnicity.

^d^Three individuals with unknown ethnicity.

The *World Report on Vision* [5] highlighted the need for inclusive and participatory leadership in order to carry out strategic planning incorporating equity by design to deliver Universal Health Coverage for eye health, and to support collaboration across governments, civil society, and the private sector. We have found that women—and in particular ethnic-minority women—are under-represented in leadership positions of IAPB member organisations, and that this disparity becomes greater at higher levels of leadership. A limitation of this study was that we were unable to ascertain whether leaders of HIC organisations were from LMICs, however, our impression is that these are very few.

IAPB member organisations are lagging behind the broader health and development sector on gender parity. For example, the cohort of 140 leading health and development organisations in the initial *Global Health 5050* cohort had female board chairs in 35.1% of organisations in 2019 [14]—twice as high as IAPB members (17.8%). Promisingly, quantifying and reporting these inequalities has proved useful in creating change, as the 2019 proportion of chairs who were women in the *Global Health 5050* organisations increased from 25.0% in the previous year.

The forthcoming *Lancet Global Health* Commission on Global Eye Health [17] will include this and other examples to highlight the need for radical action in the coming decade. Action is needed to promote and support the progression of women and people from ethnic-minority backgrounds to obtain eye health leadership positions. Increasing and sustaining this diversity is most likely if organisational efforts are embedded within a larger system—akin to *Global Health 5050*—that supports and monitors progress towards diversity goals [22, 23]. In 2018, IAPB established a *Gender Equity Working Group* to exchange good gender-responsive practices among members. More recently, the group has invited IAPB members to undertake the *Global Health 5050* self-assessment, with a view to creating targets which could then be monitored, ideally alongside some form of organisational accountability for change [22].

Beyond gender, given that many HIC organisations are primarily focused on work in LMICs, and may have significant influence on policy and funding decisions, we believe there is also a place for proactive recruitment of more internationally diverse boards with better LMIC representation. In tackling the structural barriers facing women, ethnic-minorities, and people from LMICs in these ways, global eye health organisations will be better placed to reduce the pervasive inequity in eye health.
